# Screening of antigenic epitopes related to the adhesion of the avian *Escherichia coli* Type 1 Fimbrial Agglutinin Domain

**DOI:** 10.1186/s12917-023-03742-w

**Published:** 2023-10-03

**Authors:** Junhong Chen, Wei Dai, Shengling Cui, Weiqiang Lei, Dingzhen Dai

**Affiliations:** 1 School of Animal Science and Food Engineering, Jinling Institute of Technology, Nanjing, 210038 China; 2 College of Veterinary Medicine, Yangzhou University, Yangzhou, 225009 China

**Keywords:** Type 1 fimbriae, FimH protein, Lectin domain, Antigenic epitope, Adhesion

## Abstract

**Background:**

Avian *Escherichia coli* (*E.coli*) type 1 fimbriae adhere to avian tracheal epithelial cells through the FimH protein. However, the adhesion-related antigen is still unknown. The purpose of this study was to analyze the antigenicity of the type 1 fimbrial FimH protein of wild-type avian *E. coli*, screen antigen epitopes, and prepare monoclonal antibodies (mAbs) that can block the adhesion of avian *E. coli*.

**Results:**

In this study, the nucleic acid homologies of MG2 (O11), TS12 (O18), and YR5 (O78) with K12 were 97.7%, 99.6%, and 97.7%, respectively, and the amino acid sequence similarity reached 98.7%, 99.3%, and 98.0%, respectively. The epitopes and hydrophilicities of the FimH proteins of these three strains were similar. The more obvious lectin domain epitopes were located at FimH protein positions 111–124 and 154–162. The mAbs 7C2 and 7D8 against these two epitopes were prepared. An adhesion inhibition test showed that 7C2 and 7D8 blocked bacterial adhesion to avian tracheal epithelial cells. The mAb 7C2 against the 111–124 epitope inhibited O78 strain adhesion by 93%, and the mAb 7D8 against the 154–162 epitope inhibited O78 strain adhesion by 49%, indicating that these two epitopes are closely related to the adhesion of type 1 fimbriae. However, only the 111–124 epitope-recognizing mAb 7C2 inhibited bacterial agglutination of erythrocytes, indicating that host cell receptor binding and erythrocyte agglutination are not mediated by the same spatial locations within the FimH protein.

**Conclusions:**

The results demonstrate that the mAbs 7C2 and 7D8 against FimH protein positions 111–124 and 154–162 could inhibit the adhesion of *E.coli* to the chicken trachea.

**Supplementary Information:**

The online version contains supplementary material available at 10.1186/s12917-023-03742-w.

## Background

Avian *E.coli* is a pathogen that seriously endangers the poultry industry. Its main virulence factors include fimbriae and toxins. Fimbrial adhesion enables pathogenic *E. coli* to colonize and proliferate in the body of chickens, thereby facilitating invasion [1, 2]. Type 1 fimbriae are common protruding adhesion factors that can bind to mannose-like receptors in avian airway epithelial cells through adhesion-related FimH proteins and have mannose-sensitive hemagglutination (MSHA) properties [3]. The adhesion of avian *E. coli* to airway epithelial cells helps bacteria adhere to the host’s respiratory tract, which in turn allows the bacteria to migrate and settle, eventually leading to airsacculitis, pericarditis, perihepatitis, and even sepsis, resulting in a high mortality rate and severe impact on the poultry industry [4, 5]. Fimbriae are also proteinaceous protrusions with good immunogenicity. Therefore, it is of great significance to develop fimbrial subunit vaccines for the control of avian colibacillosis. It has also been reported in the literature that avian *E. coli* type 1 fimbriae and human *E. coli* type 1 fimbriae originated from the same ancestor, and it is of public health significance to study the adhesion of the FimH protein of type 1 fimbriae to host tissue cell receptors [6, 7]. Gyimah, Panigrahy and Williams [8, 9] used extracted type 1 fimbriae to prepare a vaccine for immunizing chickens and indicated that the antibodies produced in response to immunization could greatly reduce pathogenicity. However, it is difficult to develop effective fimbrial subunit vaccines due to the lack of specific adhesion-related antigens. It is of great significance to determine the epitopes associated with adhesion of the FimH protein for screening effective immunizing antigens to prepare vaccines. Type 1 fimbriae are composed of fimbrial rods, linker subunits and the adhesion protein FimH, among which the fimbrial rods are intertwined with more than 1000 subunits encoded and expressed by *fimA*. The fimbriae are connected with FimG and FimF proteins and finally with the FimH protein, which exerts its adhesion function at the tip. The pilus rod is the main part of the fimbria, accounting for approximately 90% of the total fimbria, and each bacterium has approximately 400–1000 fimbriae [10, 11]. Although the FimH protein content is very small, it is a key component of bacterial adhesion and pathogenesis [12, 13]. Research on the efficient expression of FimH protein epitopes has important practical significance for the development of vaccines to prevent and control avian colibacillosis.

In this study, the published *fimH* gene sequence was used as a reference to design and synthesize primers to amplify and clone the *fimH* gene of wild-type *E. coli* from three different serotypes. The sequence of the amplified gene was determined, and software was used to analyze the antigenicity of the FimH protein. The antigenic epitopes were screened, and monoclonal antibodies (mAbs) against the antigenic epitopes of the FimH protein were prepared. Their ability to block adherence to epithelial cells and inhibit erythrocyte agglutination was verified, and a correlation between antigenic epitopes and adhesion and erythrocyte agglutination was observed, which provided a reference for the prevention of avian colibacillosis.

## Results

### Electrophoresis of PCR amplification products

**Fig. 1:**
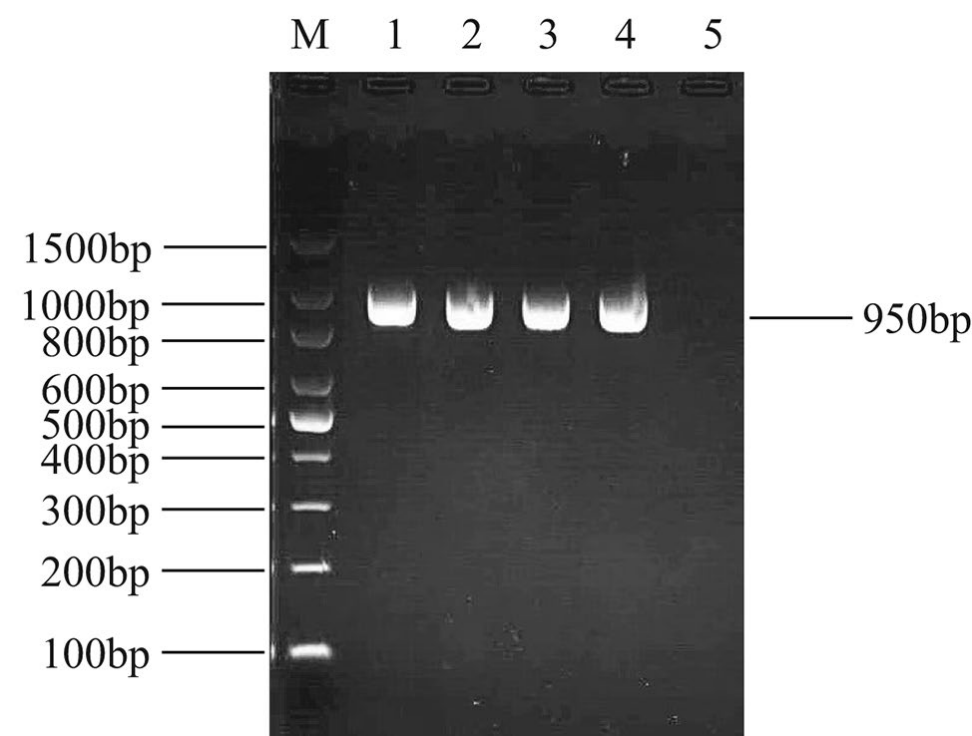
Electrophoresis results for PCR amplification products of the chromosomal *fimH* gene of four avian pathogenic *E.coli* type 1 fimbriae. M: 100 bp DNA Marker; lane 1, MG2 (O11); lane 2, TS12 (O18); lane 3, YR5 (O78); lane 4, TK3 (O1) strain as a control; lane 5, negative control. All PCR products were electrophoresed on 1.5% agarose gels

The products of three different serotypes of chicken-derived *E.coli* were amplified. A DNA band with a size of approximately 903 bp was observed, as shown in lanes 1–4 (Fig. 1), which was similar to the expected DNA size of *fimH*.

### Gene homology and amino acid sequence analysis of the *fimH* sequence

**Fig. 2:**
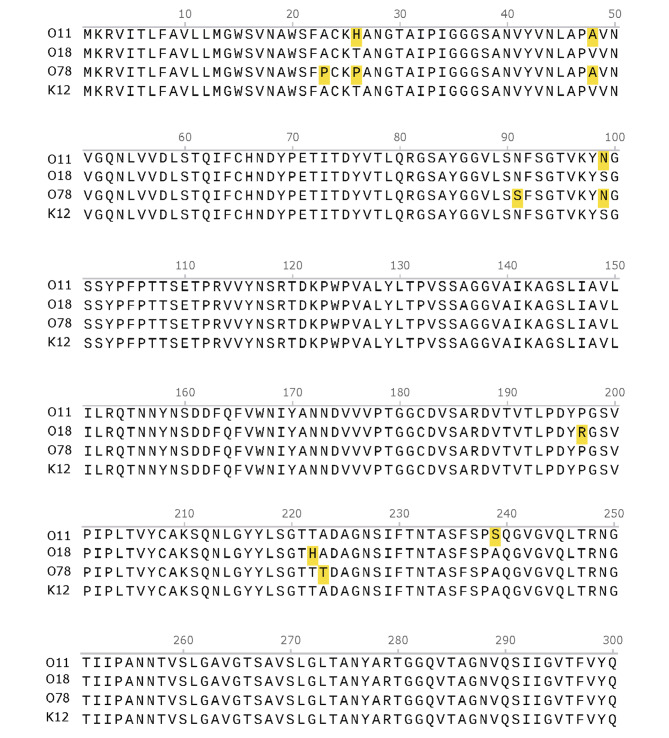
Comparison of the amino acid sequences of the FimH proteins of type 1 fimbriae from four different serotypes of avian pathogenic *E.coli*. Positions 1–21 are the signal peptide sequence. Positions 22–177 are the lectin domain, and their protein sequences are basically the same. Positions 182–300 are the pilin-binding domain, where individual amino acid variations exist. The amino acid sequence alignment of the FimH proteins of MG2 (O11), TS12 (O18), YR5 (O78), and K12 was performed using MUSCLE in SnapGene software. Amino acids that differ from the K12 amino acid sequence are highlighted in yellow

The sequences from three different serotypes of chicken-derived *E. coli*, MG2 (O11), YR5 (O78) and TS12 (O18), were analyzed by Lasergene software and compared with the *fimH* gene sequence of *E. coli* K12 type 1 fimbriae published in GenBank. In this experiment, the *fimH* gene of *E. coli* type 1 fimbriae of the O11, O18, and O78 serotypes was amplified, sequenced, and compared with the foreign K12 gene sequence; the nucleic acid homology was 97.7%, 99.6%, and 97.7%, respectively, and the amino acid sequence similarity was 98.7%, 99.3%, and 98.0%, respectively. The analysis results of the amino acid sequence encoded by the *fimH* gene are shown in Fig. 2; the amino acid sequence encoded by the *fimH* gene showed little variation.

### Prediction of FimH protein hydrophilicity

**Fig. 3:**
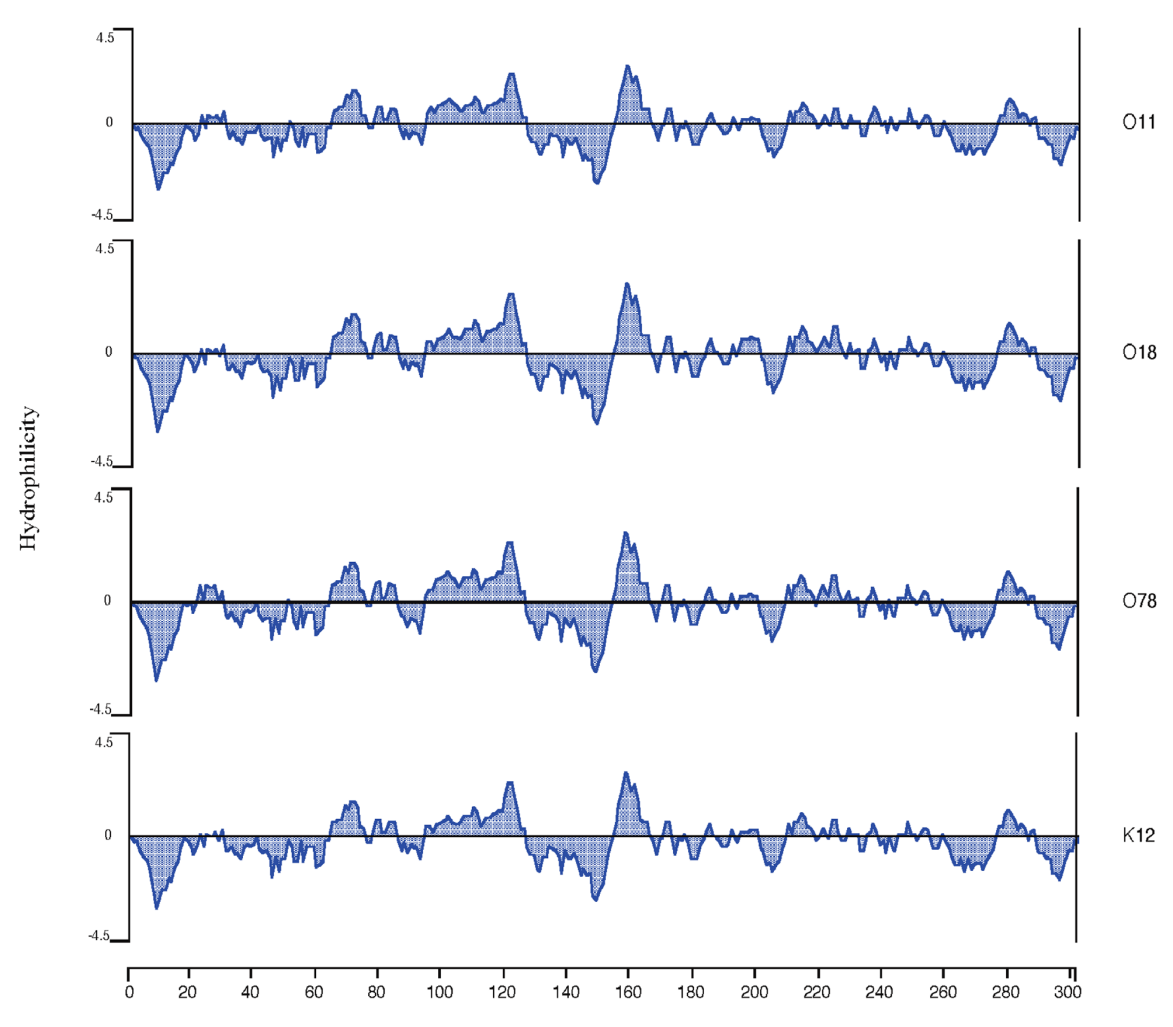
Hydrophilicity analysis of the FimH proteins of type 1 fimbriae from four different serotypes of avian pathogenic *E.coli*. The horizontal axis shows the residue number in the mature peptide; the vertical axis shows the hydrophilicity index. The hydrophilicity patterns are basically the same, and the hydrophilic regions are mainly distributed in positions 64–75, 111–126, and 154–162. The hydrophilicity patterns were generated using Protean in DNA-star software

The hydrophilicity profiles of FimH proteins from the four sources were highly similar, and the regions with stronger hydrophilicity were located at positions 67–76, 111–124, and 154–162 of the FimH protein (Fig. 3), indicating that the hydrophilic amino acids of the FimH protein of type 1 fimbriae of avian *E. coli* have similar hydrophilicities.

### Epitope prediction for the FimH protein

**Fig. 4:**
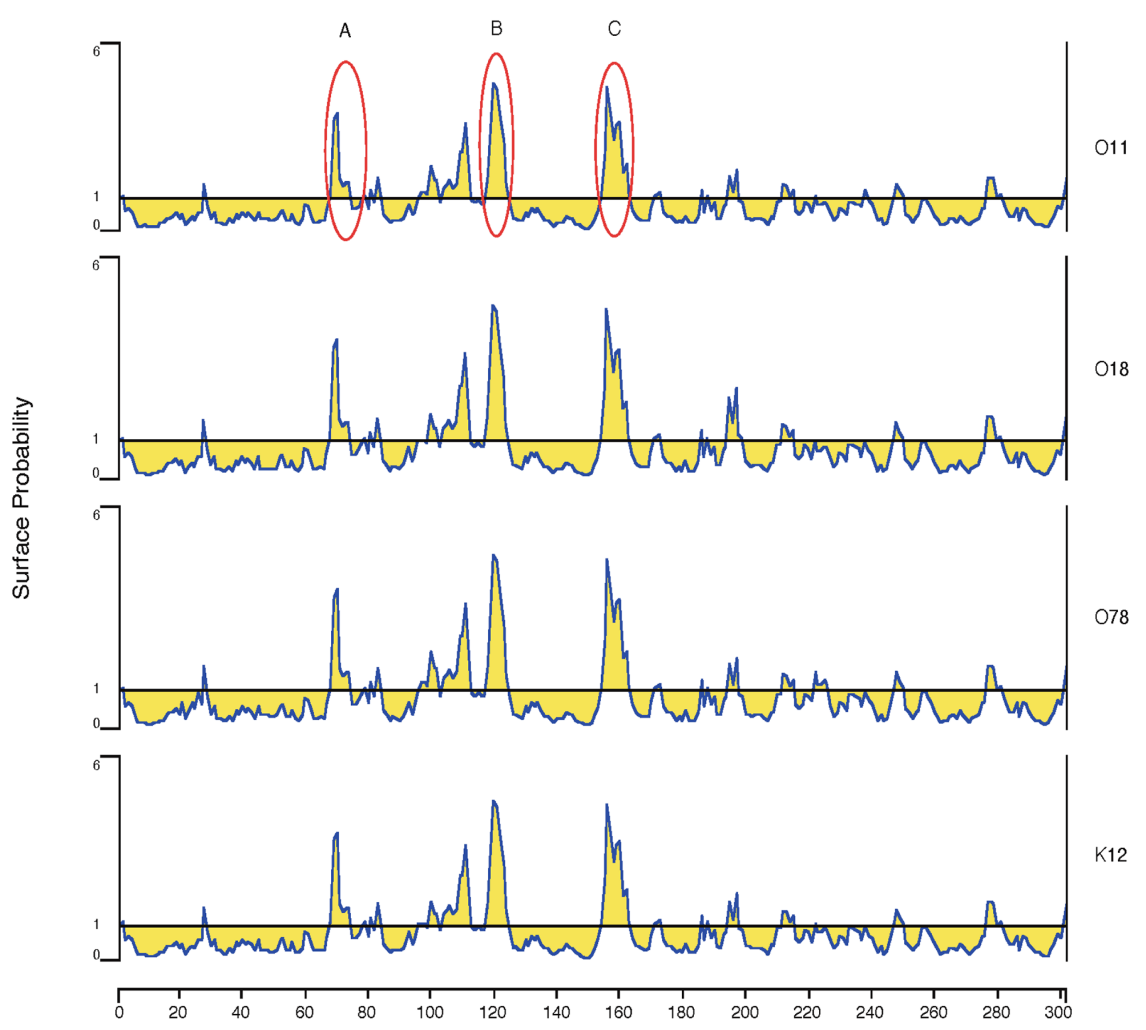
Antigenic epitope analysis of the FimH proteins of type 1 fimbriae from 4 different serotypes of avian pathogenic *E.coli*. The horizontal axis shows the residue number in the mature peptide; the vertical axis shows the surface probability plot. The antigenic epitopes are basically the same: **A** represents CHNDYPETITDY at positions 65–76; **B** represents NSRTDKPW at positions 117–124, which is the epitope for the monoclonal antibody 7C2; and **C** represents QTNNYNSDD at positions 154 to 162, which is the epitope for the monoclonal antibody 7D8. The antigenic epitope patterns were generated using Protean in DNA-star software

The epitope prediction results showed that the FimH protein epitopes of the three serotypes of avian *E. coli* type 1 fimbriae were basically the same, and there was no significant difference compared with those of the standard strain K12 (Fig. 4). A total of 3 epitope sequences were found in the FimH protein fimbrial binding domain: positions 67–76: NDYPETITDY; positions 98–124: YNGSSYPFPTTSETPRVVYNSRTDKPW, divided into two peaks, between which the NSRTDKPW peak at positions 117–124 segment was the highest; and positions 154–162: QTNNYNSDD. After alignment, the amino acid sequences of the antigenic epitopes of the four strains from different sources were found to be the same. It was hypothesized that epitopes 117–124 and 154–162 were more closely related to adhesion and could be used as candidate antigen epitopes.

### Screening and synthesis of the epitope of the FimH protein of chicken-derived *E. coli* type 1 fimbriae

According to the analysis of multiple factors, namely, hydrophilicity, specificity, and antigenicity, in this study, C-TPRVVYNSRTDKPW at positions 111–124 and LRQTNNYNSDDFQ at positions 152–164 in the amino acid sequence of the FimH protein were selected as antigenic sites. Cysteine was added to facilitate the coupling of carrier proteins. The synthesis was commissioned by Shanghai Yingjun Company to produce a polypeptide with a purity of 80.1%, which was then dissolved in ultrapure water to 1 mg·mL-1 and stored at -20 °C until use.

### Preparation of monoclonal antibodies against single epitopes

Two positive mAbs with high titers were screened against two different epitopes of the FimH protein, named 7C2 and 7D8, both of which were IgG. Among them, the mAb 7C2 had the highest titer.

### Hemagglutination inhibition test for monoclonal antibodies

The hemagglutination inhibition effects of the 7C2 mAbs against MG2 (O11), TS12 (O18), and YR5 (O78) revealed hemagglutination values of 2^6^, 2^6^, and 2^7^, respectively. The hemagglutination of the four strains was inhibited by the 7C2 mAb at dilutions of 1:2^2^, 1:2^3^, and 1:2^5^. The 7D8 mAb could not block the hemagglutination of the MG2 (O11), TS12 (O18), and YR5 (O78) strains.

### Monoclonal antibodies block *E.coli* adhesion to the trachea

**Fig. 5:**
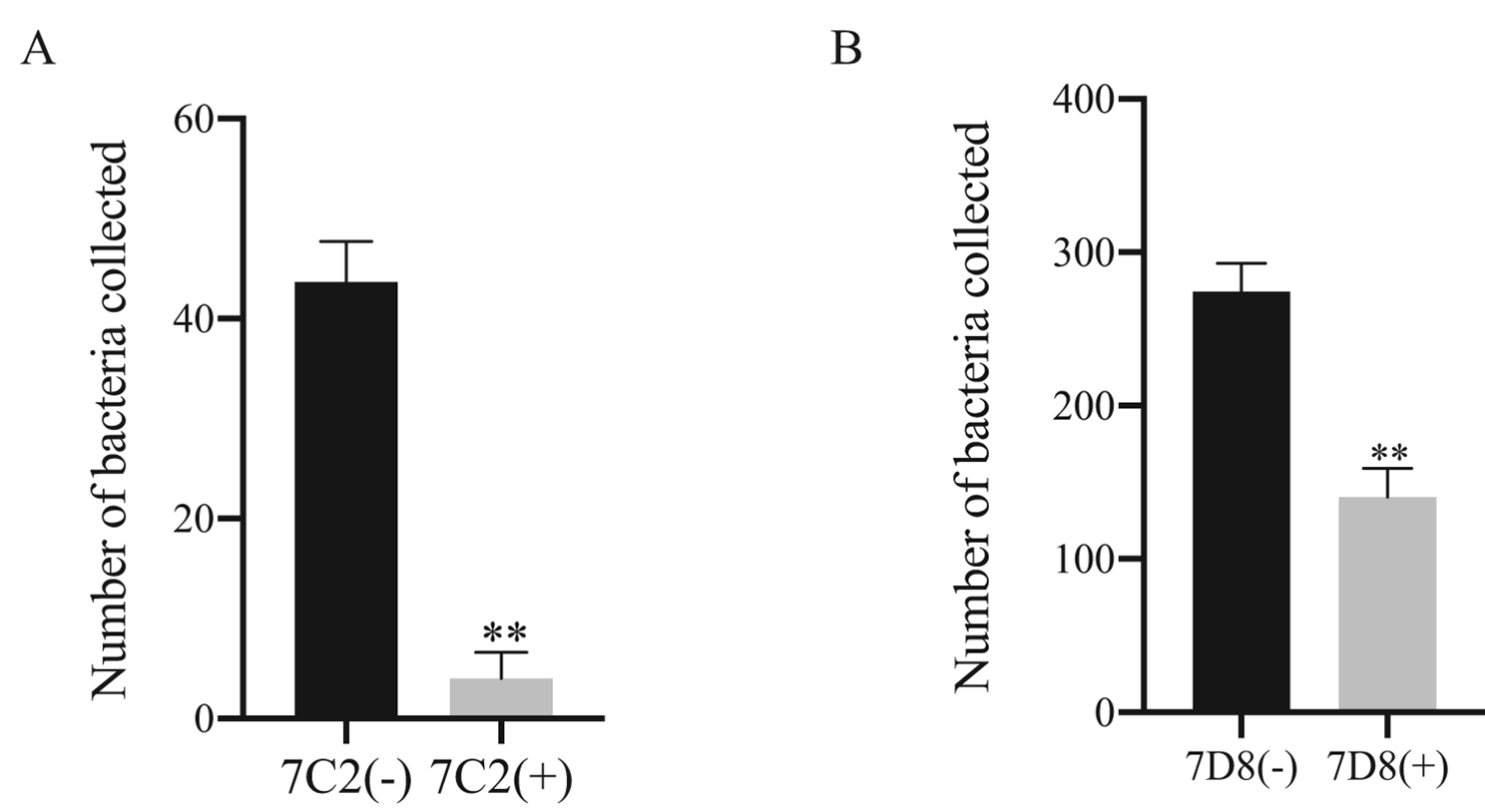
Inhibitory effect of the monoclonal antibodies 7C2 and 7D8 on fimbrial adhesion. **A** shows that the 7C2 monoclonal antibody could effectively inhibit the adhesion of bacteria to the host tracheal epithelium; **B** shows the inhibitory effect of the 7D8 monoclonal antibody, which could also significantly inhibit the adhesion of bacteria to the tracheal epithelium

The inhibition test results for the 7C2 mAb showed that the adhesion of YR5 (O78) *E. coli* could be significantly inhibited by the 7C2 mAb. Calculated based on the relative adhesion of normal bacteria set as 100%, the adhesion inhibition rate of this strain reached 93% (Fig. 5A), and the difference was extremely significant (p<0.05). The results of the inhibition test of the 7D8 mAb showed that the effect of the 7D8 mAb in inhibiting adhesion was not as good as that of the 7C2 mAb, but its adhesion inhibition rate was 49% (Fig. 5B).

### Scanning electron microscopy observation of bacterial adhesion to the trachea

**Fig. 6:**
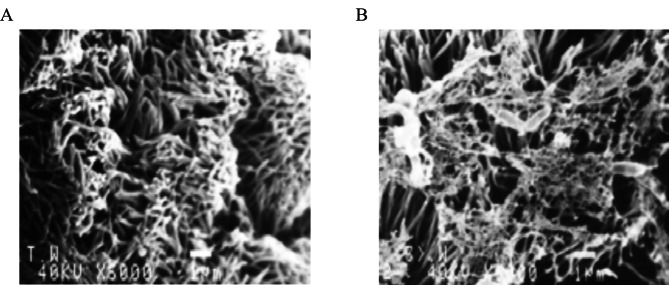
The monoclonal antibody 7C2 inhibits the adhesion of *E.coli* O78 to the tracheal epithelium in vitro. **A**, treated with monoclonal antibody; **B**, not treated with monoclonal antibody

The in vitro scanning electron microscopy results showed that *E. coli* from the bacterial suspension samples adhered to the tracheal mucosal epithelial cells on the tracheal segments in the absence of the mAb, which could be seen in any field of view on the electron microscope. However, no bacteria in the bacterial suspension adhered to the tracheal mucosal epithelial cells on the tracheal segments incubated with the mAb 7C2. Chicks were inoculated with the *E. coli* bacterial suspension without the addition of the mAb through the trachea, and bacteria adhered to the tracheal mucosa 6 h after inoculation in vivo. However, the bacterial suspension treated with the mAb showed no bacterial adhesion in the tracheal electron microscopy images after inoculation. Using scanning electron microscopy, we observed that chicken-derived *E. coli* treated with the anti-FimH protein mAb rarely attached to the chicken tracheal ciliary epithelium. The chicken-derived *E. coli* without anti-FimH protein mAb treatment was densely attached to the chicken tracheal ciliary epithelium in every field of view, indicating that the prepared mAb could significantly inhibit the adhesion of chicken-derived *E. coli* to the chicken tracheal epithelium (Fig. 6).

The above results showed that the two epitope-recognizing mAbs could specifically bind to chicken *E. coli* type 1 fimbriae, blocking the binding site of *E. coli* and tracheal mucosal epithelial cells, thereby preventing bacterial adhesion.

## Discussion

Type 1 fimbriae are adhesion factors responsible for respiratory infection by avian pathogenic *E. coli*, and most chicken-derived pathogenic *E. coli* have type 1 fimbriae. The adhesion of type 1 fimbriae is mediated by the FimH protein; that is, FimH protein adhesion is mediated by mannose, which binds to the receptor conjugates of the chicken respiratory epithelium, resulting in bacterial colonization in the chicken respiratory tract and further migration to air sacs and lungs, leading to systemic infection and thereby colibacillosis [4, 5]. Studies have shown that the FimH proteins encoded by the adhesion-related structural gene of avian *E. coli* type 1 fimbriae and human *E. coli* type 1 fimbriae share high homology. This study showed that compared with those of the human *E. coli* K12 strain, the gene sequences of FimH proteins of different avian serotypes of avian *E. coli* share high homology, the same signal peptide sequence, and a correct reading frame. Protean analysis software predicted that the hydrophilicity and antigenic epitope maps of the FimH proteins from the three serotypes were basically the same, and the amino acid sequences of the antigenic epitopes were basically the same. The nucleic acid homology of the *fimH* gene among the three chicken-derived *E. coli* type 1 fimbriae was 97.7%, 99.6%, and 97.7%, and the amino acid sequence similarity was 98.7%, 99.3%, and 98.0%. There were no differences in type 1 fimbrial adhesion gene promoter, SD sequence, or stop codon, and the fimbrial protein signal peptide was basically the same in chicken-derived and human-derived *E. coli*. This result shows that there is no obvious mutation in adhesion-related genes, and the results are consistent with other studies. This may be a protective adaptive mechanism to maintain the characteristics of adhesion to host cells, which is basically consistent with previous reports [14, 15]. Similar studies have also shown that the type 1 fimbrial FimH protein consists of two binding domains. Studies have shown that the FimH protein consists of 300 amino acids, of which the pilin-binding domain at the C-terminus is located from amino acids 162 to 299. The FimH protein is linked to FimG, and the FimG protein is linked to the FimF protein and then linked to the spring-like fimbria formed by the polymerization of FimA, thus forming the overall structure of the fimbria [15]. The N-terminal lectin domain is located in the amino acid region from positions 1 to 157 in the FimH protein, and the two binding domains are connected by a tetrapeptide linking loop. The FimH protein can bind to the avian tracheal mucosal epithelium and human urethral bladder epithelium with mannose receptors and can agglutinate red blood cells, yeast, and other cells. However, it is not clear which building blocks of the lectin domain are associated with these two roles. Schembri [16] interchanged *fimH* in type 1 fimbria-containing strains with FocH from F1C fimbria-containing strains. After removing the hydrolyzed signal peptide, among the remaining 279 amino acids, 72% of the structural units from the N-terminus of the FimH protein were retained, and these could simultaneously bind to mannose receptors and agglutinate erythrocytes. However, the remaining 56% and 66% of the structural units from the N-terminus of the FimH protein had only mannose receptor-binding ability but could not agglutinate erythrocytes. This finding showed that the receptor-binding ability and erythrocyte agglutination are related to different spatial locations of the FimH protein. In addition, artificial mutation of amino acids 77, 157, and 300 also inhibited the agglutination of erythrocytes [14, 15], which indicated that the mechanism of FimH protein-mediated agglutination of erythrocytes is complex.

Bacterial adhesion is achieved by attaching the FimH protein located on the tip of the bacterial hair to the mannose receptor on the epithelial cells of the host respiratory tract [17, 18]. Further research has found that the main structure of adhesion is a “binding bag” located in the spatial conformation of the lectin domain [19]. Pavel et al. [20] used single molecule force spectroscopy experiments to study the spatial structure of type 1 pili of *E.coli* (UPEC) infected with the human urethra. They found that the “binding bag” related amino acid sites include F1, N46, D47, N54, Q133, N135, N138, D140, D141, and F142, which are mostly hydrophilic amino acids. The “bag mouth” is a hydrophobic “tyrosine gate” composed of amino acids such as Y48, I52, and Y137, which can guide mannose into negatively charged “binding bags”, further enhancing the affinity of the “binding bags” with receptors. This affinity of FimH protein “binding bag” is common in *E.coli* type 1 fimbriae from many different sources, which depends on the strong conservation of *fimH* gene, which may be an adaptive mechanism retained by pathogens to bind to mannose receptors [17, 21]. We sequenced and analyzed the gene sequence of type 1 pili FimH protein from more than 20 APEC from different sources, and screened a group of antigenic epitopes “^132^RQTNNYNSDDFQ^143^” located at position 132–143 in the lectin domain. Further analysis showed that most of the amino acids of the epitope acted as the amino acid sites related to the “binding bag”, of which Y137 is one of the amino acids that make up the “tyrosine gate” and D140 is one of the amino acids related to receptor binding sites. In this study, the corresponding position 154–162 of the 7D8 monoclonal antibody (containing 21 signal peptide amino acids) is located in this region, which is worthy of further study.

We verified the relevance of the major epitopes in the lectin domain for receptor binding and erythrocyte agglutination. In this study, from the perspective of amino acid hydrophilicity and antigenic epitopes, for a comparative analysis of related epitopes in the lectin domain, adhesion and hemagglutination inhibition assays were performed by preparing mAbs against a single epitope of the lectin domain. As a result, two positive mAbs, 7C2 and 7D8, with high titers were screened against two different epitopes of the FimH protein. Experiments showed that the 7C2 mAb against the 111–124 epitope could agglutinate the corresponding *E. coli* containing type 1 fimbriae. At the same time, the mAb not only blocked the adhesion of bacteria to tracheal epithelial cells but also inhibited the agglutination of chicken red blood cells by *E. coli*. This result shows that the 111–124 epitope is related to the adhesion and agglutination of erythrocytes. The 7D8 mAb against positions 154–162 could inhibit the adhesion of bacteria to the chicken tracheal epithelium to a certain extent but could not prevent red blood cell agglutination, indicating that the epitope is related to the adhesion of bacteria. However, the inhibition of erythrocyte agglutination was not observed, and it is unclear whether there is a correlation; this aspect is worthy of further study. Another antigenic epitope is located at positions 67–76, but its peak value was very small. In this study, no antibody against this epitope was prepared, but its importance for adhesion needs to be further investigated. Notably, this study considered only the adhesion effect of hydrophilic amino acid epitopes, while the relationship between hydrophobic amino acids and adhesion is unclear.

In fact, studies have shown that mutation of either the receptor-binding domain or the hydrophobic amino acids of the pilus-binding domain completely abolishes hemagglutination [22, 23]. Research on the immunogenicity of the effective antigen of the FimH protein showed that blocking the adhesion of *E. coli* to host cells and the binding to mannose receptor, could effectively curb infection, providing insights for immunity-based prevention and control of avian colibacillosis [24–26].

## Conclusion

This study confirmed that the main adhesion-related epitopes of the FimH protein of chicken-derived *E. coli* type 1 fimbriae had no obvious variation. MAbs against epitopes 111–124 and 154–162 were successfully prepared, but the latter mAbs did not inhibit erythrocyte agglutination, indicating that these two epitopes are related to adhesion; however, there were differences in hemagglutination, indicating that the hemagglutination of type 1 fimbriae and adhesion to mannose receptors are not mediated by the same spatial region of the FimH protein. In view of this finding, the corresponding antigens can be obtained by constructing the expression vector of these two antigenic epitopes, and a subunit vaccine can be prepared to immunize birds and prevent avian colibacillosis. The prepared mAb can also be used as a tool to detect the adhesion of type 1 fimbriae.

## Methods

### Strains, plasmids, and experimental animals

Avian pathogenic *E. coli* MG2 (O11), TS12 (O18), YR5 (O78) isolates and TK3 (O1) [27, 28] were preserved by the Laboratory of Veterinary Microbiology, School of Animal Science and Food Engineering, Jinling Institute of Technology. Electron microscopy and MSHA assays confirmed that these three strains had type 1 fimbriae. The pMD18-T vector was purchased from Takara Company. The pMD19-T vector, pUC18 plasmid, and the recipient strains DH5α and BL21 were purchased from Nanjing Zhuyan Biotechnology Company. Eight-week-old BALB/c mice and ICR mice were provided by the Center for Comparative Medicine, College of Veterinary Medicine, Yangzhou University. Twenty-one-day-old SPF chicken embryos were provided by Nanjing Zhushun Biotechnology Co., Ltd. SP2/0 myeloma cells were purchased from Nanjing Baijiesi Biotechnology Company. The mice and the chicks were euthanized by cervical dislocation.

### Primer design and synthesis

Primers were designed by SnapGene analysis software according to the DNA sequence of the *fimH* gene published in the NCBI database. The primers were synthesized by Nanjing Sipkin Biotechnology Co., Ltd., and the size of the amplified gene fragment was expected to be 903 bp. The primer sequences were as follows: *fimH*-F 5’GGATGAAACGAGTTATTACCCTGTTTG 3’; *fimH*-R 5’ATGTCGACTGGCCTACAAAGGGCTAACGTG 3’. The primers were dissolved in ultrapure water, diluted to 25 pmol·L^− 1^, and stored at -20 ℃.

### PCR amplification, cloning, and DNA sequence determination of the target gene *fimH*

The reaction conditions were as follows: predenaturation at 94 °C for 5 min; 35 cycles of 94 °C for 30 s, 51 °C for 10 s, and 72 °C for 1 min; a final extension at 72 °C for 5 min; and 16 °C for 5 min. PCR products were analyzed by 1.5% agarose gel electrophoresis. After electrophoresis, the gel was heated and melted; the DNA was purified by a DNA purification column, collected by centrifugation, and ligated with the pTG19-T vector; and the ligation product was transformed into DH5α competent cells. Positive clones containing the *fimH* gene were obtained by screening cultures in ampicillin resistance medium and by PCR amplification identification. Plasmids were extracted and sequenced using a kit for DNA sequence determination using universal sequencing primers. The DNA sequences of the *fimH* gene of the type 1 fimbriae of the 3 different serotypes, MG2 (O11), TS12 (O18) and YR5 (O78), were determined by the same method, and the determined *fimH* gene sequences were used for bioinformatics analysis.

### Hydrophilicity and epitope analysis of the FimH protein of type 1 fimbriae

DNA-star analysis software was used, and the *fimH* gene sequences of MG2 (O11), TS12 (O18), YR5 (O78) and K12 (MG1655, NC_000913.3) were input into the system. Considering the degradation of the signal peptide of the FimH protein, the sequence of the signal peptide was removed during prediction and alignment. After translation to amino acid sequences, the hydrophilicity of the proteins was predicted, and the similarity between them was compared according to a hydrophilicity map. The measured *fimH* gene sequence was input into the software, the amino acid sequence was obtained through simulated translation, and the antigenic epitope was analyzed accordingly. The peptides with the antigenic epitope were screened and synthesized according to the above analysis results.

### Antigen preparation and animal immunization

The synthesized polypeptide was coupled to the KLH protein carrier, and the coupled polypeptide antigens for the 111–124 and 154–162 positions of the FimH protein were prepared. FimH-KLH was used as an immunogen, diluted with PBS, mixed with an equal volume of Freund’s complete adjuvant, vortexed for half an hour, and injected subcutaneously into 3 healthy female BALB/c mice for 8 weeks after complete emulsification. Each mouse was injected with 0.5 mL containing 100 µg of antigen. After 2 weeks, the second immunization was carried out in the same way with Freund’s complete adjuvant (0.25 mL, 50 µg). The third immunization was carried out 7 days after the second immunization, and 0.5 mL of incomplete Freund’s adjuvant containing 50 µg of antigen was injected intraperitoneally. One week later, blood was collected for ELISA. A booster immunization was performed with 100 µg (0.5 mL/animal) of antigen dissolved in PBS. Three days later, blood was collected for ELISA detection, and the spleen of M3 mice with the best immune response in terms of serum titer was selected for fusion.

### Screening of positive hybridoma cells

The MG2 (O11), TS12 (O18), and YR5 (O78) strains were inoculated into 500 mL of LB medium and placed in a 37 °C incubator for 72 h. After the growth of fimbriae was examined by the hemagglutination and hemagglutination inhibition test [7], the bacterial solution was centrifuged at 11,000 × g for 20 min at 4 °C. The precipitate was suspended in an appropriate amount of Tris-HCl buffer (0.05 M, pH 7.3) and then stirred at the highest speed with a magnetic stirrer for 1 h in an ice bath, and the bacterial solution was centrifuged at 11,000 × g for 20 min at 4 °C to remove bacteria and large protein complexes. The supernatant was the crude fimbrial extract. Repeated centrifugation was performed to remove bacterial residue. MgCl_2_ was added at a final concentration of 0.1 M into the supernatant. The supernatant was placed at 4 °C for 18 h and then centrifuged at 24,000 × g for 45 min at 4 °C. The pellet (fimbrial extract) was suspended in 3–5 mL of Tris-HCl buffer. Before fusion, the eyes of immunized mice were removed to collect blood to prepare positive serum, which was used as a positive control for hybridoma screening, while the serum of nonimmunized mice was used as a negative control, and a blank group was set up to adjust the values to zero. The coating concentration of antigen and the optimal dilutions of positive and negative control sera were determined by a square array test [29]. Positive clones were screened by the indirect ELISA method. The medium was changed 10 days after cell fusion, and when the hybridoma cells grew to cover 1/6 of the culture well or the culture supernatant began to turn yellow, the cells were labeled with the monoclonal antibody [12] and screened. Immunized mouse serum was used as a positive control for screening, nonimmunized mouse serum was used as a negative control, and a blank group was set up to adjust the values to zero.

### Monoclonal antibody-mediated hemagglutination inhibition test

Lyophilized chicken-derived *E. coli* was cultured in LB medium at 37 °C, passaged twice for 48 h each time, and prepared at 1 × 10^9^ CFU·mL^− 1^ with normal saline for use. Each bacterial solution was directly agglutinated with 1:100-diluted mAbs on a glass slide, and the reaction results were observed by naked eye. The presence of agglutination was judged as positive (+), and the absence of agglutination was judged as negative (-).Third-generation LB cultures of fimbriated chicken-derived *E. coli* MG2 (O11), TS12 (O18), and YR5 (O78) were centrifuged and diluted 2^− n^-fold with PBS (pH 7.4). The mAb was added to a suspension of 1 × 10^9^ CFU·mL^− 1,^ and the hemagglutination inhibition test was performed with an equal volume of 3% chicken red blood cells.

### Test of monoclonal antibody-mediated inhibition of *E.coli* adhesion to the trachea

Twenty-one-day-old SPF chicken embryos were sterilized with iodine tincture, and the chicks were removed aseptically. The trachea was removed and placed in a dish, rinsed 3 times with KRT saline, and then rinsed 3 times with 0.05 M Tris-HCl buffer. The trachea was cut into 1 × 3 mm segments and then rinsed three times with KRT saline, and 20 mL of KRT saline and 5 tracheal segments were added to each 100 mL conical flask for use. According to the method introduced by Gyimah [4], the MG2 (O11), TS12 (O18), and YR5 (O78) *E. coli* strains were inoculated into LB culture medium and cultured at 37 °C for 72 h (shaking 5–6 times during the period) before centrifugation at 2000 rpm. The pellet was washed with PBS (pH 7.4) for 2 min, which was repeated 3 times, and then the concentration of the bacterial solution was adjusted to approximately 3.0-3.5 × 10^8^ CFU·mL^− 1^ with PBS. The purified mAb was diluted 100-fold with PBS before use and temporarily stored at 4 °C for future use. One milliliter of the *E. coli* standard suspension was added to 1 mL of the diluted mAb and incubated for 1 h at room temperature with occasional shaking. One milliliter of *E. coli* standard suspension was incubated with 1 mL of PBS in the same way. The 4 mixed bacterial solutions with mAbs were added to conical flasks containing 5 tracheal segments containing 20 mL of KRT saline, and the mixed suspension of PBS without mAbs and the standard bacterial solution was used as a negative control. The cultures were incubated at 37 °C for 1 h with shaking. After removal, the tracheal segment was washed 3 times with PBS to wash off nonadherent bacteria. Then, each tracheal segment was placed in 2 mL of PBS and ground with a sterile mortar. After double dilution, 0.1 mL of sample was plated on MacConkey agar to determine the bacterial count. Using the number of bacteria collected from 5 tracheal segments incubated in PBS as the basis of adhesion, the rate of inhibition of bacterial adhesion by mAbs was calculated as follows: adhesion inhibition rate = 1- (bacterial count from samples with mAbs/bacterial count from samples without mAbs)×100%.

### Mannose adhesion inhibition test

One milliliter of the standard bacterial suspension was added to 1 mL of PBS containing 100 mg of mannose. The mixture was incubated at room temperature for 1 h with occasional shaking. Another 1 mL of bacterial suspension plus 1 mL of PBS was incubated by the same method as a control. One milliliter of the treated bacterial solution was added to a conical flask containing 20 mL of KRT solution containing 5 tracheal segments. One milliliter of untreated bacterial solution was added in the same way. Incubation was performed as described above, and the number of adherent bacterial cells was calculated.

### Scanning electron microscopy

The test was carried out according to the method of Naveh [5]. MG2 (O11), TS12 (O18), and YR5 (O78) *E. coli* cells cultured at 37 °C for 48 to 72 h were examined by electron microscopy, confirmed to have fimbriae, centrifuged, and washed. The concentration of each solution was adjusted with PBS to approximately 1 × 10^8^ CFU·mL^− 1^, and the contents of each test tube were divided into 5 mL aliquots and stored at 4 ℃ for later use. SPF chicks that were about to emerge from the shell were selected, the eggshells were sterilized, and the chicks were isolated and raised to 5 days of age after emerging from the shells. The chicks were euthanized by pentobarbital, and the upper tracheas were removed aseptically, washed in PBS, cut longitudinally into 10 × 3 mm segments, rinsed three times with PBS, and then temporarily stored in PBS for future use. The prepared YR5 (O78) bacterial suspension was removed, and 1 mL of prediluted mAb was added to each tube. At the same time, 1 mL of PBS was added to the negative control group. After mixing, the mixture was placed at room temperature for 1 h so that the mAb and bacteria were fully functional. Five tracheal segments were placed in each tube and incubated at 37 °C for 30 min on a shaker, and the tracheal segments were removed and washed three times with PBS to wash away unadhered bacteria. Each tracheal segment was fixed with 1% glutaraldehyde for 2 h, rinsed with PBS, dehydrated in 35%, 55%, 75% and 100% acetone, and then placed in an HCP-2 desiccator (product of Hitachi). After critical point drying, a gold film was plated in an IB-5 ion sputtering apparatus (made in Japan), and then the samples were observed and imaged by scanning electron microscopy.

In vivo test: Five-day-old chicks were inoculated with 0.1 mL of the above *E. coli* solution (concentration 10^8^ CFU·mL^− 1^) through the trachea using a syringe with a smooth needle tip, and some chicks were sacrificed 6 and 12 h after inoculation. The trachea was dissected aseptically, treated the same as in the above in vitro test, and examined by scanning electron microscopy. In addition, the tracheal epithelium of chickens without bacteria was used as a control, and the same method was used.

### Electronic supplementary material

Below is the link to the electronic supplementary material.

Supplementary Material 1

## Data Availability

The datasets analyzed during this study are available from the corresponding author on reasonable request. The datasets generated and/or analysed during the current study are available in the NCBI repository, [the *fimH* gene sequences of K12 accession number: NC_000913.3. (https://www.ncbi.nlm.nih.gov/nuccore/NC_000913.3/), MG2 (O11) GenBank accession number: OR351927 (https://www.ncbi.nlm.nih.gov/nuccore/OR351927.1/), TS12 (O18) GenBank accession number: OR351926 (https://www.ncbi.nlm.nih.gov/nuccore/OR351926.1/) and YR5 (O78) GenBank accession number: OR351928 (https://www.ncbi.nlm.nih.gov/nuccore/OR351928.1/)].
