# Supplementary figures and images for: Screening of antigenic epitopes related to the adhesion of the avian Escherichia coli Type 1 Fimbrial Agglutinin Domain
Source: BMC Vet Res. 2023 Oct 3;19:187. doi: 10.1186/s12917-023-03742-w (PMC10546689; doi:10.1186/s12917-023-03742-w)

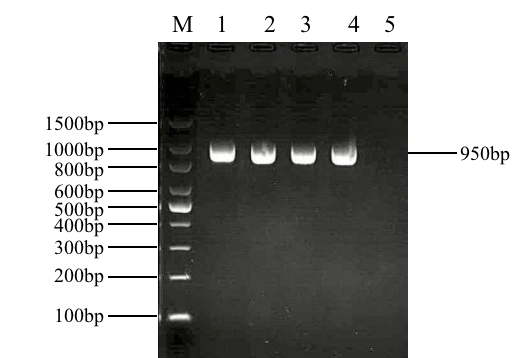
fig.1

original full-length gels images


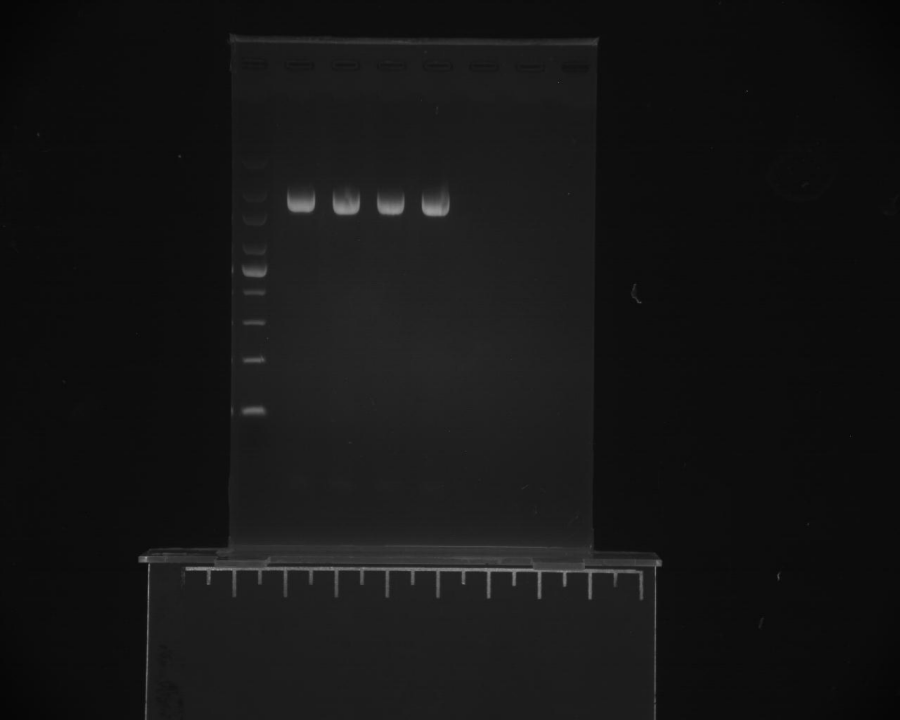

Supplement: Supplementary file 1 — Supplementary Material 1 [file 12917_2023_3742_MOESM1_ESM.doc]
